# Left ventricular pseudoaneurysm secondary to recurrent mitral prosthetic valve endocarditis

**DOI:** 10.1002/ccr3.6522

**Published:** 2022-11-04

**Authors:** Hironobu Nishiori, Goro Matsumiya

**Affiliations:** ^1^ Department of Cardiovascular Surgery Chiba University Hospital Chiba Japan

**Keywords:** infective endocarditis, left ventricular pseudoaneurysm, mitral valve replacement, prosthetic valve endocarditis

## Abstract

An 86‐year‐old man who had undergone two mitral valve replacements developed heart failure due to prosthetic valve infection and left ventricular pseudoaneurysm (LVPA). LVPA due to infective endocarditis is rare and caused by the abscess formation in the left ventricular myocardium. Infective endocarditis caused by enterococci requires attention to relapse.

## Abstract 2

Left ventricular pseudoaneurysm (LVPA) caused by prosthetic valve endocarditis is a rare condition that results from the abscess formation in the left ventricular myocardium. Infective endocarditis caused by enterococci requires attention to relapse.

## 1 CASE

**FIGURE 1:**
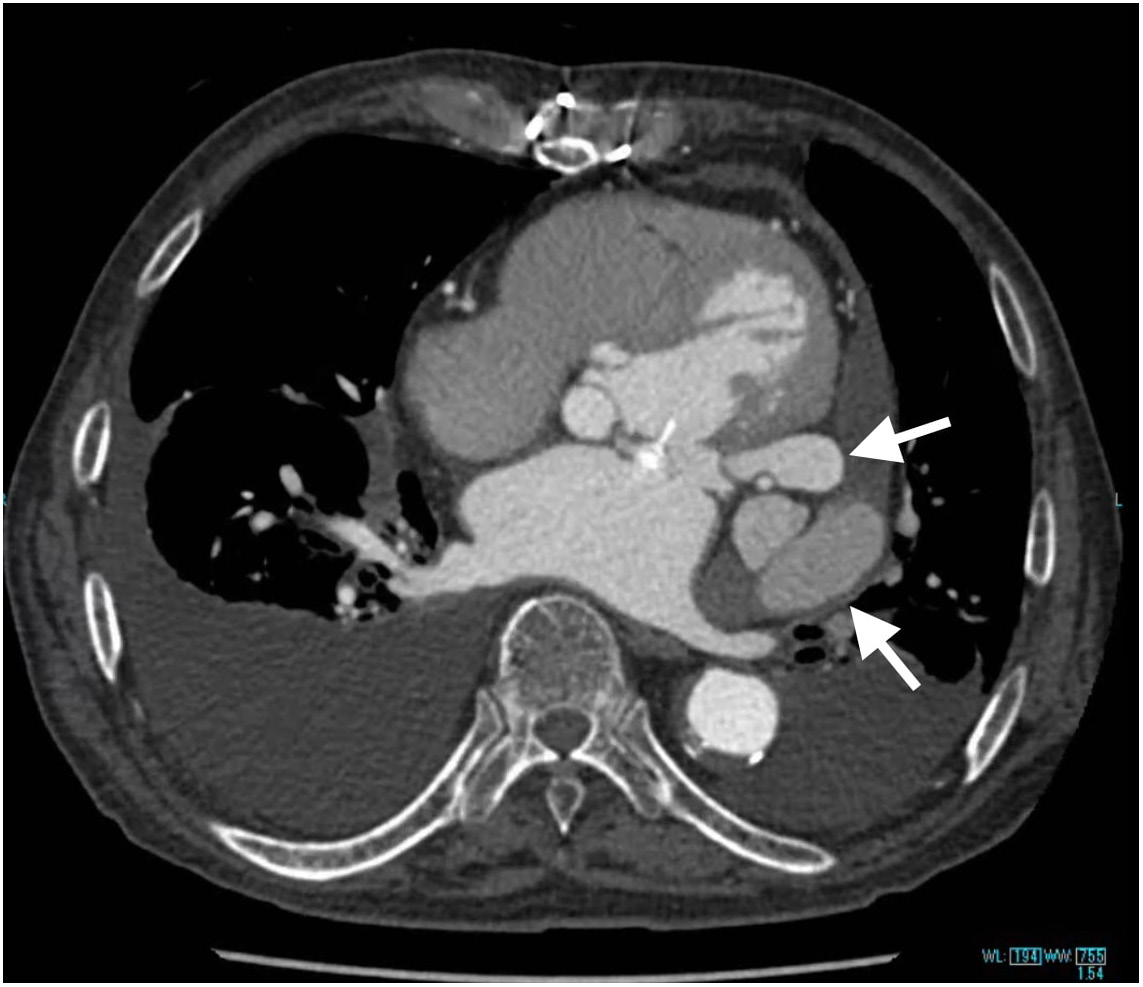
Coronal view of computed tomography imaging showing the pseudoaneurysm at the posterior wall of the left ventricular (arrow)

**FIGURE 2:**
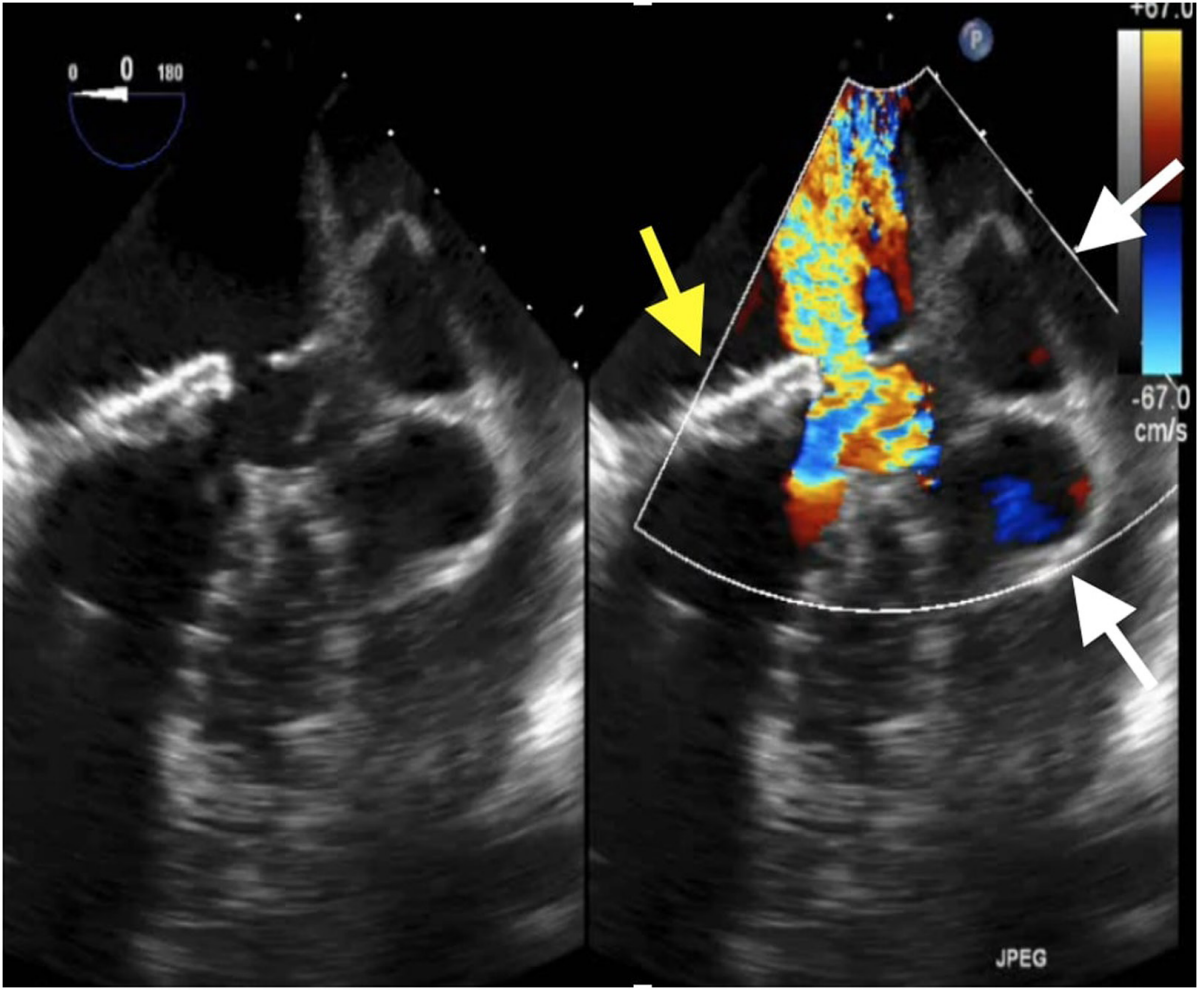
Transesophageal echocardiography showing the partially detached mitral prosthetic valve with mitral valve regurgitation (yellow arrow), and left ventricular pseudoaneurysm with color Doppler flow (white arrow)

**FIGURE 3:**
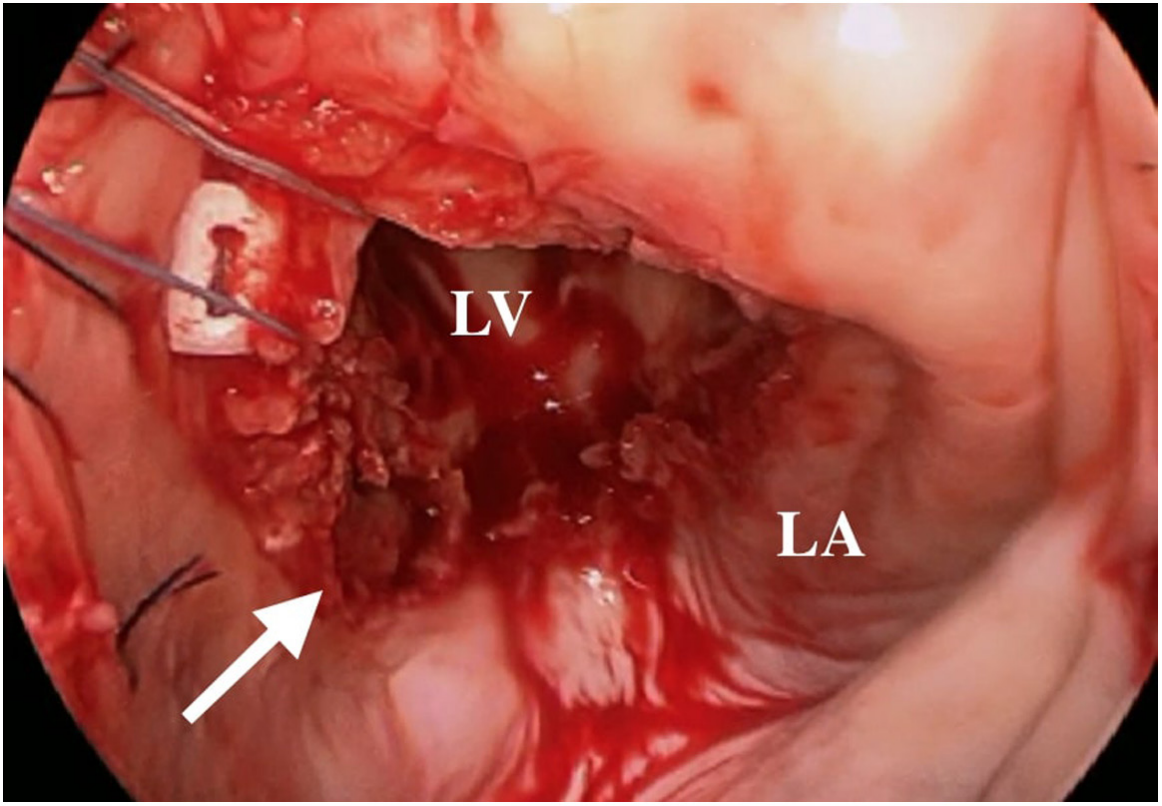
The intraoperative imaging from the left atrium showing the orifice of the pseudoaneurysm at the posterior side of the mitral annulus (arrow). LV, left ventricular; LA, left atrium

**FIGURE 4:**
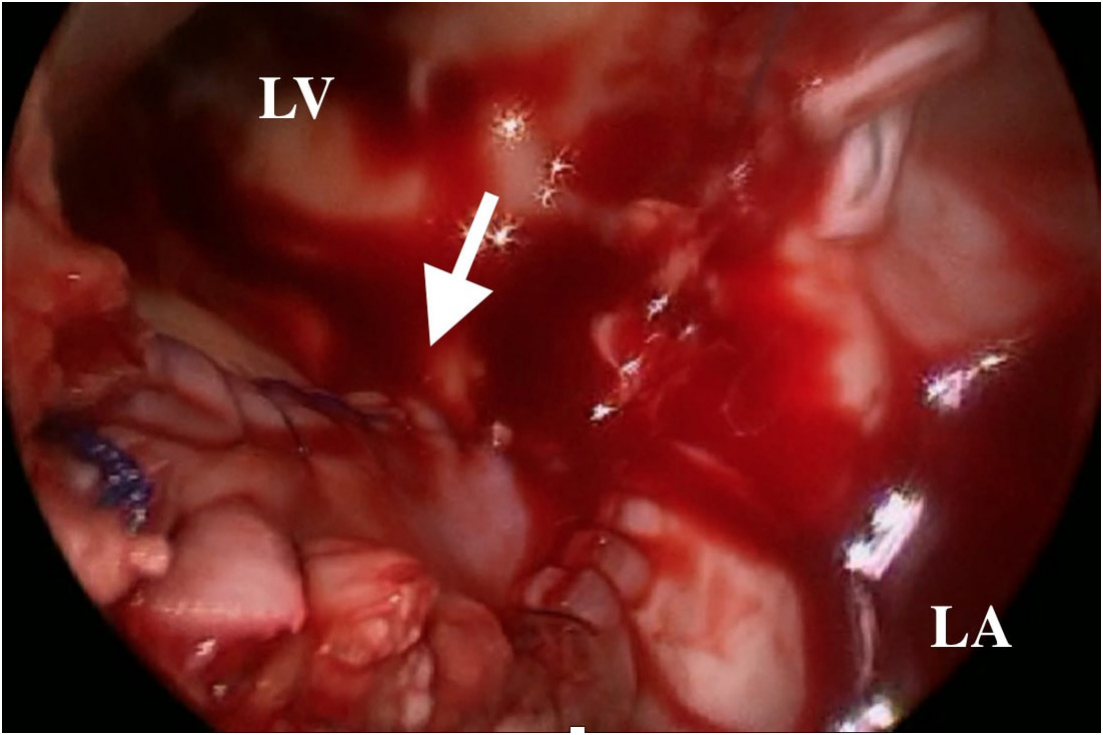
The intraoperative imaging from the left atrium showing the closed orifice of the pseudoaneurysm using hemashield patch (arrow). LV, left ventricular; LA, left atrium

An 86‐year‐old man with a history of mitral valve replacement (MVR) for infective endocarditis 17 years ago and re‐do MVR for prosthetic valve endocarditis 4 years ago presented with fever and shortness of breath. The blood cultures were positive for Enterococcus spp, and antibiotic therapy with Piperacillin/Tazobactam was initiated. The computed tomography imaging showed the pseudoaneurysm formed at the posterior wall of the left ventricular (Figure 1). Transthoracic echocardiography exhibited the partially detached prosthetic valve from the mitral annulus with severe para‐valvular leakage (Figure 2). An oval‐cut hemashield patch was continuously sutured to the LVPA orifice, closing the LVPA. (Figures 3 and 4) Then, MVR was performed. The patient was treated with six‐week course of intravenous antibiotics, followed by lifelong oral antibiotics. The 1‐year follow‐up echocardiography showed no mitral regurgitation or blood flow into the LVPA.

Left ventricular pseudoaneurysms (LVPAs) due to mitral valve infective endocarditis are rare, accounting for <1% of all LVPA, and are fatal with a 35–40% risk of rupture.^1^ LVPA is formed when an abscess invades the left ventricular myocardium forming an abscess cavity and predisposing to left ventricular wall dissection.^2^ Abscess formation is more likely to occur with prosthetic valve endocarditis, and enterococcus etiology is one of the risk factors for infective endocarditis relapse.^3^ It should be noted that infective endocarditis caused by enterococci can recurrently relapse, as in this case, leading to LVPA formation due to mitral annular abscess after MVR.

## AUTHOR CONTRIBUTIONS

Hironobu Nishiori: Cared for the patient and got the patient consent form and prepared the clinical picture and computed tomography imaging data, and wrote the report. Goro Matsumiya: Read and approved the final version of the report.

## FUNDING INFORMATION

None.

## CONFLICT OF INTEREST

None declared.

## CONSENT

We have obtained the consent of the patient for publication.

## Data Availability

None.
